# PP87 How Innovative Is The Innovation? Early Experience In Assessing The Innovativeness Of Emerging Medical Technologies In Singapore

**DOI:** 10.1017/S0266462325103048

**Published:** 2025-12-29

**Authors:** Wing Fai Sofie Ng, Zhen Long Ng, Hong Ju, Hua Pey Gan, Swee Sung Soon

## Abstract

**Introduction:**

The assessment of a technology’s innovativeness, which indicates its capacity to transform practice, is crucial for strategic planning in health care. However, clear assessment criteria are often lacking. This abstract presents the early experience of the Agency for Care Effectiveness with the UK Government’s Medical Technology Innovation Classification Framework to identify innovative medical technologies (MedTechs) anticipated to disrupt clinical practice in Singapore.

**Methods:**

ACE’s horizon scanning methodologies were employed to identify innovative MedTechs, based on innovation programs of regulatory bodies, such as the United States Food and Drug Administration breakthrough device designation. The UK Framework was applied, using only the technology aspects (novelty) and not the implementation elements (expected shift in clinical practice) to assess the novelty of MedTechs. Three evaluators independently classified each MedTech as incremental (exists within healthcare system), transformative (novel application of existing technology), or disruptive (first-in-class) based on desktop research. The usability of the classifications was assessed through user experience and inter-rater agreement, with consensus reached for most disagreements.

**Results:**

Among 228 innovative MedTechs identified based on regulatory body designations, pre-consensus inter-rater agreement was 76 percent across innovation categories, with higher consistency (89%) for incremental innovations but lower agreement (64%) for transformative and disruptive categories. The inconsistencies stemmed from limited understanding of the use of MedTechs and their expected shift in local clinical practice. Following consensus, 42 percent were classified as incremental and 58 percent as transformative or disruptive. To enhance future usability, transformative and disruptive technologies were combined into a single highly innovative category to expedite categorization. Highly innovative technologies were then prioritized for local clinician input.

**Conclusions:**

The UK Framework offers a valuable approach to assess the level of innovativeness of MedTechs. By combining transformative and disruptive technologies into a highly innovative category, ACE could expediently prioritize impactful MedTechs to inform the system on upcoming innovations with disruptive potential. This fit-for-purpose approach could offer resource-constrained health technology assessment bodies a means to assess the innovativeness of MedTechs.

